# The analysis of rural revitalization serviceplatform in smart city under back propagation neural network

**DOI:** 10.1371/journal.pone.0317702

**Published:** 2025-03-18

**Authors:** Gongyi Jiang, Weijun Gao, Meng Xu, Mingjia Tong

**Affiliations:** 1 Foreign Languages Department, Tourism College of Zhejiang, Hangzhou, China,; 2 Faculty of Environmental Engineering, The University of Kitakyushu, Kitakyushu, Japan,; 3 Innovation Institute for Sustainable Maritime Architecture Research and Technology, Qingdao University of Technology, Qingdao, China,; 4 Faculty of Chemical Engineering and Technology, Zhejiang University of Technology, Hangzhou, China; Marshall University, UNITED STATES OF AMERICA

## Abstract

To achieve rural revitalization and enhance the development of rural tourism, this study employs a back propagation neural network (BPNN) to construct a rural revitalization development model. Additionally, the Grey Relation Analysis (GRA) algorithm is used to classify rural revitalization efforts across different cities. Consistency testing is applied to analyze rural revitalization indicators, and a tourism service evaluation model is established to assess rural revitalization tourism services from the perspective of smart cities. The research results indicate that: (1) the training results and expected values of the ten cities are relatively consistent, and the classification of rural revitalization development is good; (2) The five major indicators of tourism information services, tourism security services, tourism transportation services, tourism environment services, and tourism management services all meet the consistency test, and the consistency test results are all less than 0.1, confirming the reliability and effectiveness of the research data; (3) The tourism information and management services are mainly evaluated at level C, accounting for 62% and 62.5% respectively. The tourism transportation and safety services are mainly evaluated at level D, and the model can indicate the level of rural revitalization tourism service; (4) Compared with other algorithms, the GRA-BPNN algorithm performs the best in rural revitalization evaluation, with an accuracy of 92.3%, precision of 91.8%, recall rate of 93.7%, and F1 score of 92.7%. This study optimizes the rural revitalization tourism service platform, enhances the quality of rural tourism, promotes the development of the rural tourism industry, and contributes to the realization of rural revitalization.

## 1. Introduction

### 1.1 Research background and motivations

With the rapid advancement of information technology, smart cities have become a core strategy for promoting sustainable urban development. Smart cities not only enhance a city’s overall competitiveness but also foster economic and social progress, paving the way for a green, intelligent, and sustainable future [1,2]. However, the development of smart cities is not confined to urban areas; the construction of smart villages, as an extension of smart city initiatives, is gradually becoming a crucial component of rural revitalization strategies. By leveraging digital and information technologies, smart villages can effectively improve the allocation of rural production resources, promote their rational use, and facilitate coordinated economic, social, and environmental development in rural areas [3]. One of the core objectives of the rural revitalization strategy is to narrow the urban-rural divide and promote comprehensive rural development [4]. Among these efforts, rural tourism, as a key area for driving economic growth and increasing farmers’ income, plays an indispensable role in advancing rural revitalization [5].

With the progress of China’s new urbanization initiatives, the focus has shifted from urbanization rates to the quality of urban-rural transformation. This shift makes the coordination between rural revitalization and new urbanization a critical means for achieving sustainable development across urban and rural areas [6]. In recent years, the application of information technology, big data, and intelligent solutions in rural revitalization has gained increasing importance, offering significant potential to support the social, economic, and environmental sustainability of rural areas [7]. Against this backdrop, Liu et al. (2021) examined the role of urban financial systems in improving credit frameworks for towns and proposed financial measures to stimulate agricultural innovation, improve the financial well-being of rural residents, and support the implementation of rural revitalization strategies [8]. Similarly, Zhang et al. (2022) developed a model for poverty alleviation risk monitoring and analysis using the back propagation neural network (BPNN) and the natural breakpoint method, providing effective decision-making support [9]. Furthermore, Guo et al. (2022) quantitatively analyzed the coupling mechanism between technological innovation and rural revitalization, offering scientific recommendations for advancing agriculture and rural development [10]. Zhao et al. (2020) demonstrated that big data technology played a critical role in optimizing e-commerce, smart agriculture, and rural governance platforms, thereby advancing rural revitalization [11]. Other studies have focused on key challenges in implementing rural revitalization strategies. Jun (2020) identified labor, land, and economic factors as crucial drivers of rural revitalization and argued that labor attraction and land reforms could significantly facilitate the strategy’s implementation [12]. Chen (2021) explored the application of big data and internet technologies in traditional rural industries, highlighting how the rise of the livestreaming economy provided fresh momentum for rural revitalization [13]. Additionally, Cen et al. (2022) utilized the entropy method to analyze the impact of the digital economy on rural revitalization, emphasizing the intermediary role of industrial upgrading driven by the digital economy [14]. Within the context of integrating smart cities and rural revitalization, the intelligent development of rural areas is seen as an important pathway for narrowing the urban-rural gap and fostering the development of smart villages. Building smart villages can enhance rural infrastructure and environmental development while promoting the rapid growth of smart tourism [15,16]. Zhu et al. (2022) demonstrated through intelligent design and field demonstration of smart village living circles that smart environments could effectively boost rural tourism development, thereby driving comprehensive rural economic revitalization [17]. Yu et al. (2020) examined the construction of smart tourism in characteristic towns, highlighting its advantages in promoting rural economic and environmental development. They also analyzed existing challenges, aiming to optimize smart tourism and advance rural revitalization efforts [18].

However, although these studies provide theoretical support and practical guidance for various aspects of rural revitalization, most of the research primarily focuses on macro-level issues such as policy frameworks, resource allocation, and industrial transformation. There is a noticeable lack of in-depth investigation into specific areas, particularly the optimization of rural tourism services. Addressing this gap, the present study proposes an optimization method for a rural revitalization service platform based on the BPNN. The aim is to leverage intelligent methods to enhance the quality of rural tourism services and provide new decision-making support for rural revitalization. As one of the earliest neural networks to be extensively studied and applied, BPNN is characterized by its simplicity in structure, ease of understanding, and implementation, making it particularly suitable for solving small- and medium-scale problems [19]. In the field of rural revitalization, BPNN has demonstrated robust predictive capabilities. For instance, Du and Zhao (2023) compared BPNN with the Analytic Hierarchy Process (AHP) and proposed that BPNN’s self-learning and high-performance attributes made it highly effective in predicting outcomes within the agricultural sector of rural revitalization [20]. Wu et al. (2020) highlighted BPNN’s efficiency and accuracy in evaluating land ecological security within rural revitalization efforts [21]. To further enhance the evaluation capabilities of rural tourism service platforms, this study incorporates the Grey Relation Analysis (GRA) algorithm. GRA is particularly effective in situations with incomplete or uncertain information, as it analyzes the relationships among system factors to reveal their intrinsic connections and developmental trends [22]. By integrating BPNN with GRA, the approach not only improves prediction accuracy but also enables a more comprehensive evaluation of the quality of tourism services in the context of rural revitalization.

### 1.2 Research objectives

To improve the level of rural tourism, promote its development, and achieve rural revitalization, firstly, BPNN is utilized to construct a training model, and integrates GRA algorithm to classify the development of rural revitalization; Secondly, consistency testing is used to analyze the indicators of rural revitalization, comparing the confidence interval (CI), composite reliability (CR), and eigenvalues; Finally, a tourism service evaluation model is implemented using BPNN to evaluate and analyze the rural revitalization tourism situation from the perspective of smart cities. The comparison with the Generative Adversarial Network (GAN), Convolutional Neural Network (CNN), and Long Short-Term Memory (LSTM) and other algorithms in rural revitalization evaluation confirms the accuracy of GRA-BPNN. This study presents a method that integrates BPNN with GRA, offering significant innovation and improvements compared to previous research. Prior studies have primarily focused on leveraging BPNN for data prediction and analysis. While BPNN exhibits strong predictive capabilities, it often faces limitations when dealing with complex, multidimensional relationships, especially under conditions of incomplete or uncertain information. In contrast, by incorporating GRA, this study effectively addresses BPNN’s shortcomings in handling uncertainty and complexity. GRA reveals the intrinsic relationships among factors and enables accurate evaluation of tourism service quality, even in the presence of incomplete information, thereby enhancing the model’s reliability and accuracy. Previous research has largely concentrated on the construction of smart villages, rural revitalization, and the application of big data technologies. Although these studies provide valuable theoretical support at a macro level, they often neglect the specific issue of optimizing service quality in rural tourism. This study focuses on this specific area by combining BPNN and GRA, not only providing a comprehensive evaluation of rural tourism service quality but also enhancing decision-making support during the implementation of rural revitalization strategies. Notably, this research employs consistency tests to analyze rural revitalization indicators, comparing confidence intervals, composite reliability, and eigenvalues, thereby offering more precise data support for practical applications. The innovation of this study is further reflected in the optimization of its decision-support functionality. While previous research has mainly emphasized the overall development of smart villages, with limited detailed analysis in the tourism service domain, this study enhances the precision of decision-support systems by integrating BPNN and GRA. This combination not only improves the predictive accuracy of rural tourism service quality but also provides precise data support for the concrete implementation of rural revitalization strategies, facilitating their execution. In summary, this study demonstrates clear innovation and advantages over prior research by improving the precision of service quality evaluations, addressing gaps in rural tourism research, and providing accurate decision-making support. These contributions significantly advance the practical application of rural revitalization strategies.

## 2. Literature review

NN was widely applied in rural revitalization. Juan & Hsu (2022) used Artificial Neural Network (ANN) model to predict the development of rural revitalization, confirmed the relationship between the characteristics of different regions and the development of rural land transfer, and contributed to the promotion of rural revitalization [23]. Muhammad & Lee (2019) found that ANN could predict the land use situation and land cover change in northern Sumatra, Indonesia, to promote rural land reform, development, and revitalization [24]. Based on the theory of rural typology, Zhou et al. (2021) regionalized China’s rural types with cluster analysis. They discovered that different paths of rural revitalization were different, indicating the complexity and difficulty of rural revitalization [25]. Lu & Bao (2022) established a dynamic evaluation model of rural logistics development by using Fuzzy Neural Network (FNN), Moran index, and kernel density estimation technology and explained the overall situation of rural logistics development through the model. It was significant in achieving rural revitalization [26]. Sun et al. (2023) believed that computer virtualization played an essential role in improving the rural environment, promoting all-round construction and development of rural areas, advancing the rural economy, and structural optimization of various industries, to realize rural revitalization [27]. Li et al. (2021) used NN technology to classify new and old rural buildings, thereby improving rural monitoring capabilities. It was of great significance for understanding the development of new rural areas and provided a new direction for realizing rural revitalization [28]. Zhang & Zhang (2020) studied the use of the ANN method to identify rural types, and trained the NN model with different sample data. The influence of the model on the results of rural type identification can be analyzed, which was significant for the scientific differentiation of villages and the reasonable realization of rural revitalization [29]. Hou et al. (2021) used deep learning (DL) and artificial intelligence technology to realize the informatization of rural banks, and the integration of NN models can realize the data prediction and processing of rural bank customer information. These improved the growth of rural economy, elevated rural informatization, promoted rural governance, and provided a new idea for realizing rural revitalization [30]. The development of rural tourism was one of the effective ways to achieve rural revitalization, and scientific evaluation of tourism land was the premise for optimizing the layout of rural tourism industry. Xian et al. (2021) employed NN to construct an evaluation model and analyzed the limiting factors of the competitive development of rural tourism land, which made a certain contribution to promoting rural revitalization [31]. Gao et al. (2023) adopted the NN model to analyze rural industry development’s influencing factors and expected income. They discovered that tourism, infrastructure and transportation planning accounted for a relatively high weight in rural industrial resource utilization, of which tourism accounted for the highest proportion and was the core of rural revitalization industry development [32].

Previous studies focused more on traditional rural revitalization concepts, without fully considering the impact of digitization and intelligence on rural revitalization service platforms, especially in the comprehensive evaluation of rural revitalization development from the viewpoint of smart cities. There was a lack of universal research on rural revitalization service platforms under different geographical, cultural, and economic conditions. This study introduced the perspective of smart cities and considered the impact of digitization and intelligence on rural revitalization service platforms, making up for the shortcomings of previous research in this field. Through the comprehensive application of BPNN, GRA algorithm, consistency testing, and tourism service evaluation model, this study attempted to achieve a comprehensive evaluation of rural revitalization. This helped to have a more comprehensive understanding of the current situation and potential problems of rural revitalization. In addition, it provided a new theoretical framework and empirical basis for promoting the development of rural revitalization service platforms and made important contributions to achieving sustainable development of rural revitalization.

## 3. Research methodology

### 3.1 Smart city theory

**Fig 1:**
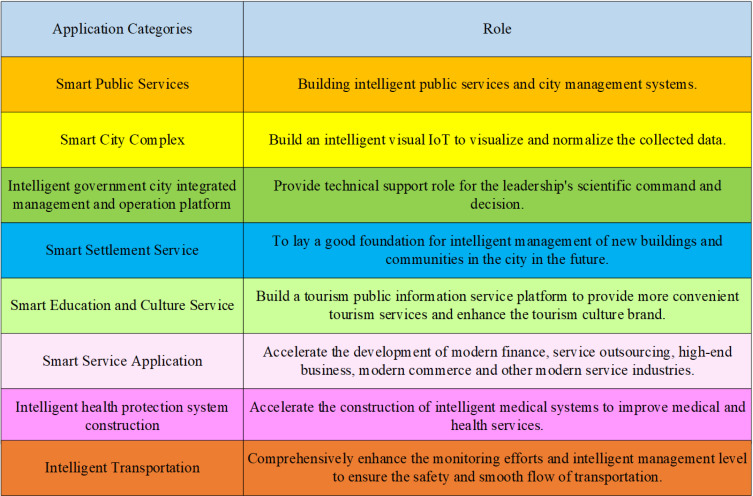
Classification of smart city application projects.

The smart city indicates the use of various IT or innovative concepts such as the internet, cloud computing, and big data in urban planning, design, construction, management, and operation, to enhance the efficiency of urban resource utilization, optimize urban management and services, and improve citizens’ quality of life [33]. Smart city has two driving factors: one is the internet, cloud computing, and other IT; the other is the open urban innovation ecology gradually nurtured in the knowledge society environment [34]. There are many application projects for smart cities, including eight aspects. The specific classification is presented in Fig 1.

### 3.2 Characteristics of rural revitalization

**Fig 2:**
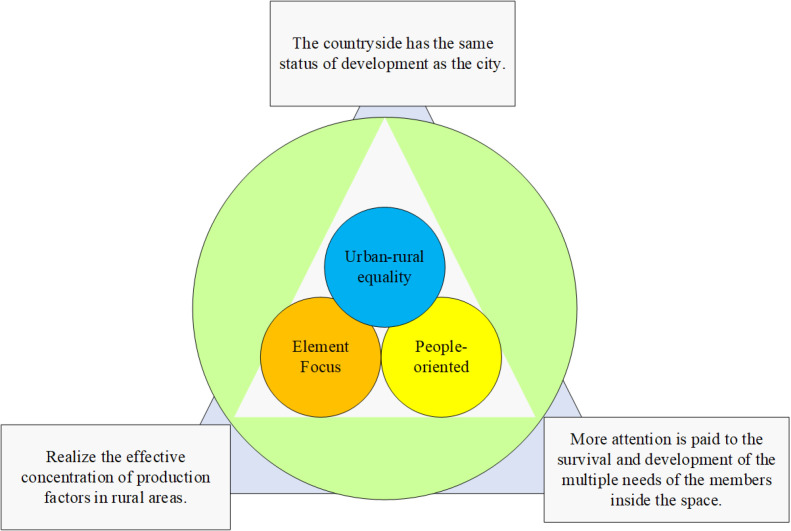
Three core features of the rural revitalization strategy.

Rural revitalization requires prioritizing the development of agriculture and rural areas [35]. It is a major decision made at the 19th National Congress of the Communist Party of China and a major historical task for comprehensively building a modern socialist country [36]. The rural revitalization strategy has three core features urban-rural equality, element focus, and people-oriented [37]. The specific core features are shown in Fig 2.

### 3.3 Smart village theory

**Fig 3:**
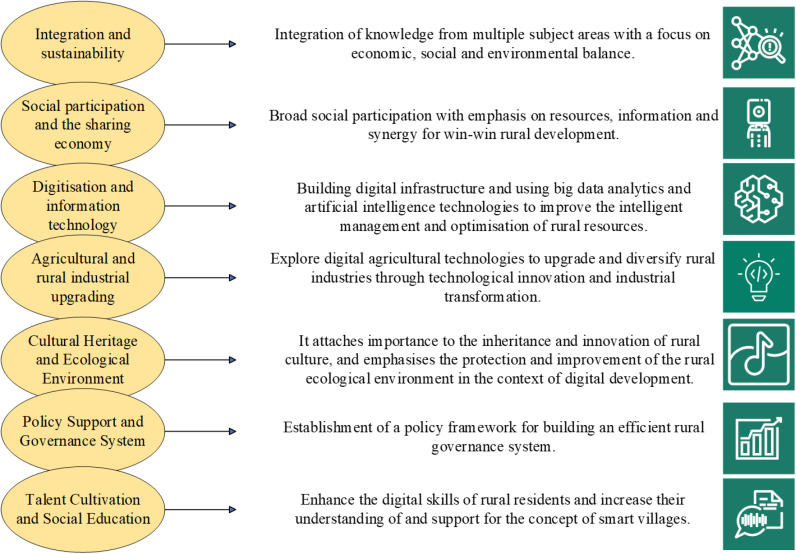
The construction principles of the basic framework of smart village theory.

Smart village refers to the use of cloud computing, big data, Internet of Things, and other technologies to build a system application platform serving government management, agricultural income increase, people’s livelihood happiness, etc., to integrate basic social information, industrial development, environmental monitoring, and other elements. In this way, intelligent life value systems such as rural internal affairs management and promoting people’s livelihood services can be realized [38]. The construction of smart villages can improve the efficiency of rural economic development, accelerate the improvement of social governance, and promote the improvement of rural residents’ living standards [39]. The construction principles of the basic framework of smart village theory are depicted in Fig 3.

### 3.4 Application of NN

NN, which belongs to machine learning and DL, also known as ANN, has the ability to mimic the way biological neurons transmit signals to each other [40]. NNs can be classified into different types. The common ones are the CNN and Recurrent Neural Network (RNN) [41]. CNN is usually used for image recognition, computer vision processing, or pattern recognition. RNNs can adopt time series data to make predictions about future outcomes.

### 3.5 Construction of the BPNN model

This study selects the BPNN as the foundational model, favoring it over more complex modern machine learning techniques based on several considerations. Compared to other advanced algorithms, BPNN is a neural network model that is structurally simple, computationally efficient, and easy to implement, making it particularly suitable for problems involving relatively small to medium-sized datasets. In practical applications such as rural revitalization, data often lack the scale and high dimensionality required by DL. Thus, using BPNN not only ensures efficient computation but also avoids the heavy dependence on large datasets and substantial computational resources typically associated with DL models. Additionally, BPNN’s self-learning capability allows it to automatically optimize weights and adapt to different types of data and patterns, reducing the complexity of manual adjustments. This adaptability is especially critical in fields like rural revitalization, where data can exhibit significant variability. Furthermore, compared to more complex models such as deep CNN or LSTM, BPNN offers better interpretability. This is particularly important for policymakers, who require a clear understanding of the model’s predictions and underlying mechanisms to make informed decisions. Finally, while modern machine learning techniques may outperform in handling more complex applications, rural revitalization scenarios often involve smaller datasets. In such cases, BPNN achieves satisfactory predictive performance with less training data and lower computational costs. Therefore, considering its balanced advantages in accuracy, computational efficiency, and interpretability, BPNN emerges as a practical and reasonable choice for addressing rural revitalization challenges.

**Fig 4:**
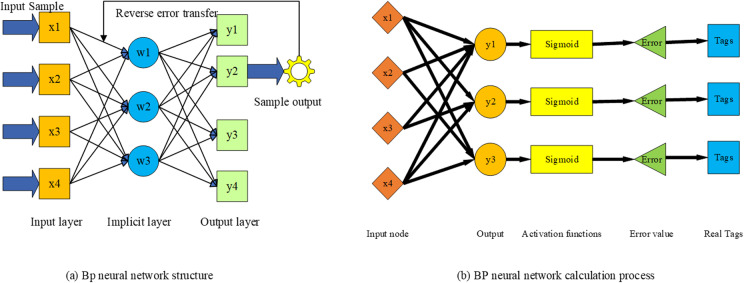
BPNN structure.

BPNN is a multilayer feedforward neural network (FNN) trained using the BP algorithm. It was proposed by Rumelhart and McClelland in 1986 [42]. BPNN has several layers of hidden units between the input and output layers, which have no direct contact with the outside world and can affect the relationship between the input and output layers [43]. The BPNN structure is demonstrated in Fig 4.

The calculation process of BPNN consists of a forward and a reverse calculation. In the forward propagation process, each layer of neurons only affects the state of the next layer of neurons. In the backpropagation process, error signals will be returned along the original connection path by adjusting the weights of each neuron [44].

To update weights and biases, the gradient descent method can be employed, optimizing network parameters by calculating the gradient of each neuron. The equation for calculating the gradient of a single neuron using the Sigmoid function reads:


$$Y_{1}=\alpha \left(w_{1}\left(X\right)+\beta_{1}\right)$$ (1)

$Y_{1}$and $w_{1}$ represent the output and weight of the first hidden layer; *X* refers to the number of input nodes; $\beta_{1}$ means the deviation; *t* stands for the actual value. The calculation of the loss function U is:


$$U=\frac{1}{2}{\left(Y_{1}-t\right)}^{2}$$ (2)

If the gradient of the fully connected layer has multiple neurons, the loss function is calculated as follows:


$$U=\frac{1}{2}\overset{j}{\underset{k}{\sum }}{\left(Y_{1k}-t_{k}\right)}^{2}$$ (3)

When passing in the forward direction, the $B_{mn}$ is set to the weight between nodes *m* and *n*; $O_{m}$ is the threshold value of node *m*; $V_{m}$ is the output node value. The calculation of the output value of each node $D_{n}$ is written as equation (4):


$$D_{n}=\overset{x-1}{\underset{m=0}{\sum }}B_{mn}V_{m}+O_{m}$$ (4)

During backpropagation, the activation function’s formula is derived based on the form of the activation function. For instance, the gradient calculation under the Sigmoid activation function in backpropagation for a BPNN is shown in Equation (5):


$$f\left(x\right)=\frac{1}{1+e^{-x}}$$ (5)

*x*represents the input value, and $f\left(x\right)$ denotes the activation function’s output, mapping the input *x* to a range between (0, 1). The Sigmoid function compresses each input to a range of 0 to 1, making it suitable for binary classification outputs or hidden layers. The derivative of the Sigmoid activation function is expressed as follows:


$$f^{\prime }\left(x\right)=f\left(x\right)\cdot \left(1-f\left(x\right)\right)$$ (6)

$f\left(x\right)$represents the Sigmoid activation function’s output, while $f^{\prime }\left(x\right)$ is its derivative, indicating sensitivity to the input value *x*. The derivative of the Sigmoid function reflects the rate of change at a specific input. It reaches its maximum when the input value is near 0 and approaches 0 when the input value diverges towards positive or negative infinity. This characteristic underpins the vanishing gradient problem in backpropagation.

During backpropagation, the gradient of each parameter is calculated to update network weights using gradient descent. The gradient for each weight is computed as shown in Equation (7):


$$\frac{\partial e}{\partial w}=\delta \cdot x$$ (7)

$\frac{\partial e}{\partial w}$represents the gradient of the loss function *e* with respect to the weight *w*. This gradient is used to adjust network weights to minimize the loss. *δ* is the error term, indicating the magnitude of the output error, and *x* is the value input to the neuron. The error term *δ* is derived from the difference between the actual output *y* and the target output *t*, as calculated in Equation (8):


$$\delta =\left(y-t\right)\cdot f^{\prime }\left(x\right)$$ (8)

$f^{\prime }\left(x\right)$is the actual output of the neural network, and *t* is the target value.

The objective function used in this study is the mean square error (MSE) used to measure the difference between the model output and the actual target value. The equation for calculating the MSE is as follows:


$$MSE=\frac{1}{m}\overset{m}{\underset{i=1}{\sum }}{\left(Y_{i}-\overset{´}{Y_{i}}\right)}^{2}$$ (9)

*m*represents the sample size, $Y_{i}$ refers to the actual target value, and $\overset{´}{Y_{i}}$ indicates the target value predicted by the model.

Initially, GRA is used to filter the rural revitalization indicators, calculate their relational degree, and optimize the structure of the input and hidden layers in the BPNN based on the desired output. This process allows for a more precise evaluation of key service factors in rural revitalization. This study employs the GRA algorithm to simplify rural revitalization indicators, primarily because GRA effectively handles situations with small datasets or data lacking typical patterns. Its advantages include independence from specific statistical assumptions and its ability to remain effective even when information is incomplete or indicators deviate from standards. Specifically, GRA selects the optimal value as a benchmark and dynamically calculates the relational degree between each evaluation object and the optimal value, objectively reflecting relationships within the data. Unlike dimensionality reduction techniques such as principal component analysis (PCA), GRA can handle nonlinear relationships and small datasets without requiring strict preprocessing or assumptions about the data. Through relational degree analysis, GRA identifies key indicators with a significant impact on the research objective while excluding low-relevance indicators, thereby enhancing the model’s specificity and effectiveness.

## 4. Experimental design and performance evaluation

### 4.1 Datasets collection

The data selected in this study are from the National Bureau of Statistics (NBS), China National Environmental Monitoring Centre (CNEMC), and the statistical yearbook of each city, and the data range is from 2000 to 2021. NBS covers agriculture, production, economy, education, and other aspects. It is a database with wide coverage and high data authority. CNEMC undertakes the task of national environmental monitoring and leads the development of environmental monitoring technology. It provides monitoring information, reports, and technical support for national environmental management and decision-making, and gives technical guidance for national environmental monitoring work. The statistical yearbook of each city is a part of the NBS, which mainly contains the authoritative rural agricultural data of each city.

**Fig 5:**
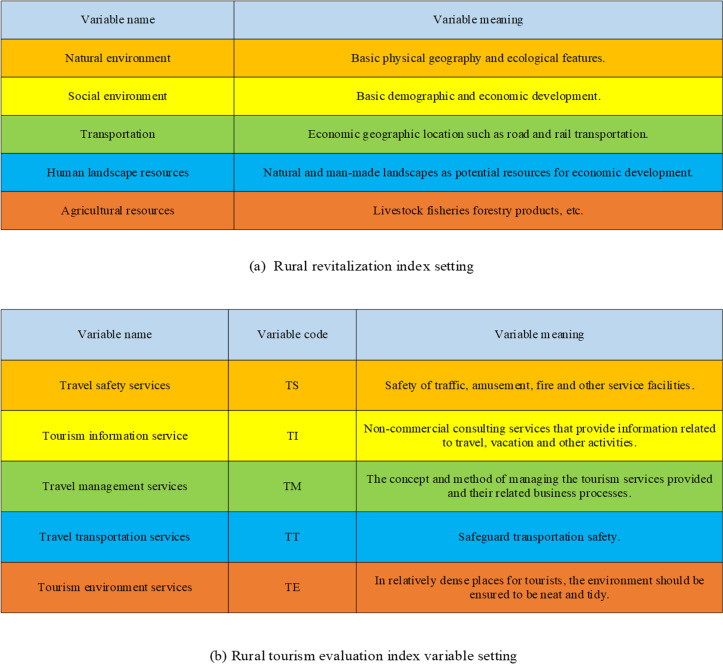
Setting of specific indexes.

The setting of specific indexes of rural revitalization is suggested in Fig 5.

Fig 5 illustrates the specific indicator settings for rural revitalization and rural tourism evaluation, which are crucially related to the inputs of this study’s model. In Fig 5(a), the rural revitalization indicators include the natural environment, social environment, transportation, cultural and scenic resources, and agricultural resources. These indicators comprehensively cover various aspects of rural development, reflecting the overall status of rural revitalization. The natural environment and cultural and scenic resources provide the foundation for tourism development, while the social environment and transportation highlight the potential and accessibility of rural development. Agricultural resources represent the core of the rural economy. In Fig 5(b), the rural tourism evaluation indicators include tourism safety services, tourism information services, tourism management services, tourism transportation services, and tourism environment services. These factors directly influence the quality of the tourism experience and the development of the tourism industry. Tourism safety and transportation services ensure visitors’ safety and convenience, information services enhance visitor satisfaction, management services improve service quality, and environmental services maintain cleanliness in the tourism environment. These indicators, selected through the GRA algorithm by excluding low-relevance indicators, are used as input variables for the BPNN model. High-relevance indicators ensure the validity and relevance of the input data, enabling the model to more accurately reflect the actual situation of rural revitalization and predict future trends. This setup not only improves the model’s precision but also provides scientific support for decision-makers, aiding in resource optimization, enhancing the quality of rural tourism services, and ultimately advancing rural revitalization.

As the raw data may contain missing values, duplicate records, or outliers, this study implements rigorous data cleaning procedures to filter and correct all data. Missing values are handled using the mean imputation method, while outliers are identified and corrected through comparisons with historical data or industry standards, ensuring data accuracy and reliability. To address inconsistencies in the dimensions of different indicators, the study utilizes the premnmx function in MATLAB for normalization. This process scales all input data to the range [-1, 1], eliminating potential biases caused by differing units of measurement and ensuring that all variables exert equal influence in the model. During the preprocessing phase, feature selection is conducted by employing the GRA method to evaluate the correlation of each indicator. Indicators with a correlation below 0.5 are removed to reduce data complexity, minimize noise effects on the model, and improve data validity and accuracy. After normalization and feature selection, the study further standardizes the data to ensure consistency in scale and distribution. Additionally, a thorough review of data completeness is conducted to ensure that no critical variables are omitted and that no logical errors exist. These data preprocessing steps ensure high-quality input data, eliminating potential biases and enhancing the efficiency of model training and the accuracy of predictions, thereby laying a solid foundation for subsequent BPNN model training and analysis.

### 4.2 Experimental environment

The computer system used in this study runs a 64-bit Windows 10 operating system, with an Intel i7-8700K processor, 8GB of RAM, and a 256GB solid-state drive (SSD). Matrix Laboratory (MATLAB) version 9.11 is employed for model training and simulation. MATLAB, widely used in data analysis, DL, and signal processing, is chosen as the primary tool for this research due to its efficient numerical computation capabilities. Given the limited hardware resources in the experimental environment, particularly constraints in memory and processing power, challenges such as computation delays or memory shortages may arise when handling computationally intensive tasks. To address these issues, this study employs techniques such as data preprocessing, normalization, and feature selection to reduce computational demands. Additionally, the model structure is appropriately simplified during its design phase to enhance operational efficiency within the hardware constraints.

During computations, the 8GB of memory and Intel i7-8700K processor configuration are sufficient for basic data analysis and model training tasks. However, for larger datasets or more complex model computations, further optimizations are necessary. These include reducing the training sample size, adjusting model parameters, or leveraging distributed computing to improve efficiency. The fast read and write speeds of the SSD also contribute to enhanced data loading and storage efficiency, ensuring the model can be trained and simulated effectively. Overall, despite the limitations in computational resources, the study ensures successful model training and simulation by optimizing resource allocation and algorithms, ultimately achieving the expected research outcomes.

### 4.3 Parameters setting

This study uses the GRA algorithm to simplify the indicators of rural revitalization from City 1 to City 10. By excluding indicators with a correlation degree below 0.5, raw data of tourism information services, tourism safety services, tourism transportation services, tourism environment services, and tourism management services with high correlation with other indicators are selected as input values for the BPNN model.

The study utilizes a BPNN model with parameter optimizations to improve the accuracy and reliability of rural revitalization level predictions. The input layer is configured with 16 neurons, representing 16 key indicators to encompass multiple aspects of rural revitalization, such as tourism information, transportation, safety, and environment. These indicators are selected using the GRA algorithm to filter highly relevant data, thereby reducing redundancy and improving model efficiency. The output layer consists of a single neuron to predict the overall level of rural revitalization. The hidden layer is set to include 10 neurons, a number determined through experimental validation to balance model complexity and predictive capacity while avoiding overfitting or underfitting. During training, the error threshold is set to less than 0.00094 to ensure high precision, with 1,830 iterations conducted to allow the model to learn thoroughly and approximate the optimal solution. Compared to other commonly used methods, the BPNN demonstrates significant advantages in capturing the nonlinear relationships among input data, enhancing computational efficiency, and speeding up the training process. Additionally, it provides clear and interpretable outputs. Consequently, the BPNN model in this study achieves high accuracy in predicting rural revitalization levels and provides robust support for policymaking and decision-making in rural revitalization, showcasing its innovation and effectiveness in this field of research.

The BPNN training model has a significant promoting effect on the development of rural tourism. By using multiple indicators related to rural revitalization as inputs, covering tourism information, safety, transportation, environment, and management services, BPNN can learn and analyze the complex relationships between these factors. By training historical data, the model can predict the future trend of rural revitalization, offer a scientific basis for decision-makers, and enable them to more accurately formulate and adjust rural tourism development strategies. In addition, the advantage of BPNN lies in its ability to discover nonlinear relationships and patterns in the data, thereby gaining a more comprehensive understanding of the impact of diverse indicators on rural tourism development. Through this approach, the BPNN training model provides refined and personalized guidance for rural tourism, helping to optimize resource allocation, improve service quality, and ultimately promote the sustainable and prosperous development of rural tourism.

### 4.4 Performance evaluation

**Fig 6:**
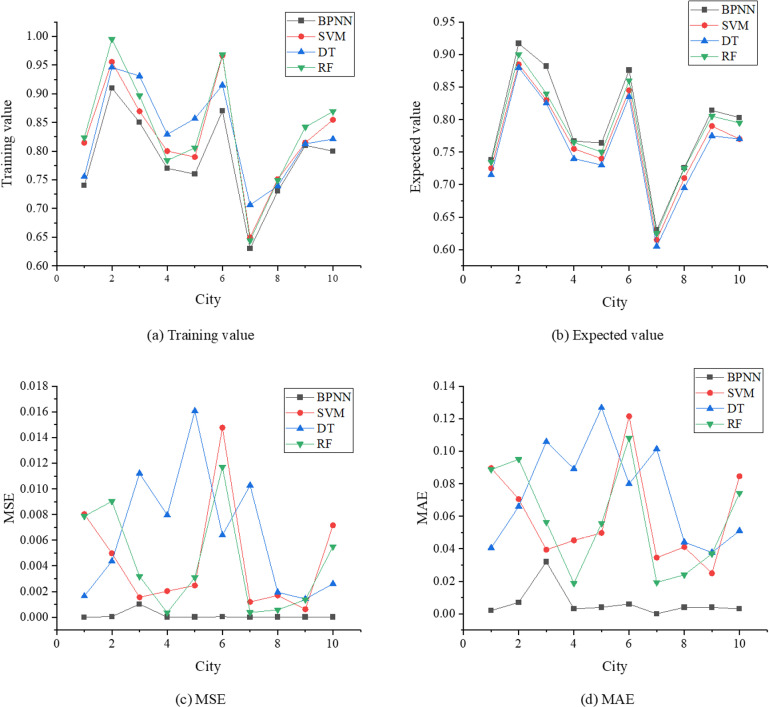
Results of performance comparison between BPNN model and common benchmark models.

This study compares the performance of the BPNN model with common baseline models, including Support Vector Machine (SVM), Decision Tree (DT), and Random Forest (RF). The evaluation metrics used are Mean Squared Error (MSE) and Mean Absolute Error (MAE). The specific results are presented in Fig 6.

In Fig 6, the BPNN model demonstrates superior performance compared to other baseline models in most cases, particularly excelling in error control. Specifically, the BPNN achieves consistently lower MSE and MAE values, indicating a smaller discrepancy between the expected values and training results. Notably, in cities 1, 2, 5, and 7, the expected values are closely aligned with the training results, with MSE values in the 10^−6^ range and relatively small MAE values, showcasing the model’s efficiency. In contrast, the SVM model exhibits slightly lower predictive accuracy, especially in cities 6 and 10, where the MSE values reach 0.0148 and 0.0072, respectively, and the MAE values are relatively large, at 0.1215 and 0.0846. These results indicate significant prediction errors for certain cities when using the SVM model. The DT and RF models show even less stability. For instance, the DT model reports high MSE values in several cities, particularly in cities 3 and 5, where the values are 0.0112 and 0.0161, respectively. These values are accompanied by correspondingly large MAE values, suggesting potential inaccuracies in handling certain datasets. While the RF model performs slightly better than DT, its prediction accuracy is still inferior to BPNN. Although RF achieves lower MSE and MAE values in some cities, such as cities 4 and 7, it exhibits higher error values in others, such as cities 2 and 6. In summary, the BPNN model achieves the best performance in this study, with higher predictive accuracy and lower error rates. Although SVM, DT, and RF can complete prediction tasks, their results show significant deviations in certain cases. Using MSE and MAE as evaluation metrics effectively highlights the differences in the practical performance of various models, further validating the superiority of the BPNN model in addressing the research problem.

Additionally, the training results and expected values for cities 1, 4, 5, and 6 are approximately 0.7, indicating a “good” level of rural revitalization development. For cities 2, 3, 6, 9, and 10, these values exceed 0.8, signifying an “excellent” level of rural revitalization. Meanwhile, in city 7, both the training results and expected values hover around 0.6, indicating an “average” level of development. Overall, the training results align closely with the expected values across all ten cities, demonstrating the model’s capability to represent varying levels of rural revitalization development among different cities accurately.

**Fig 7:**
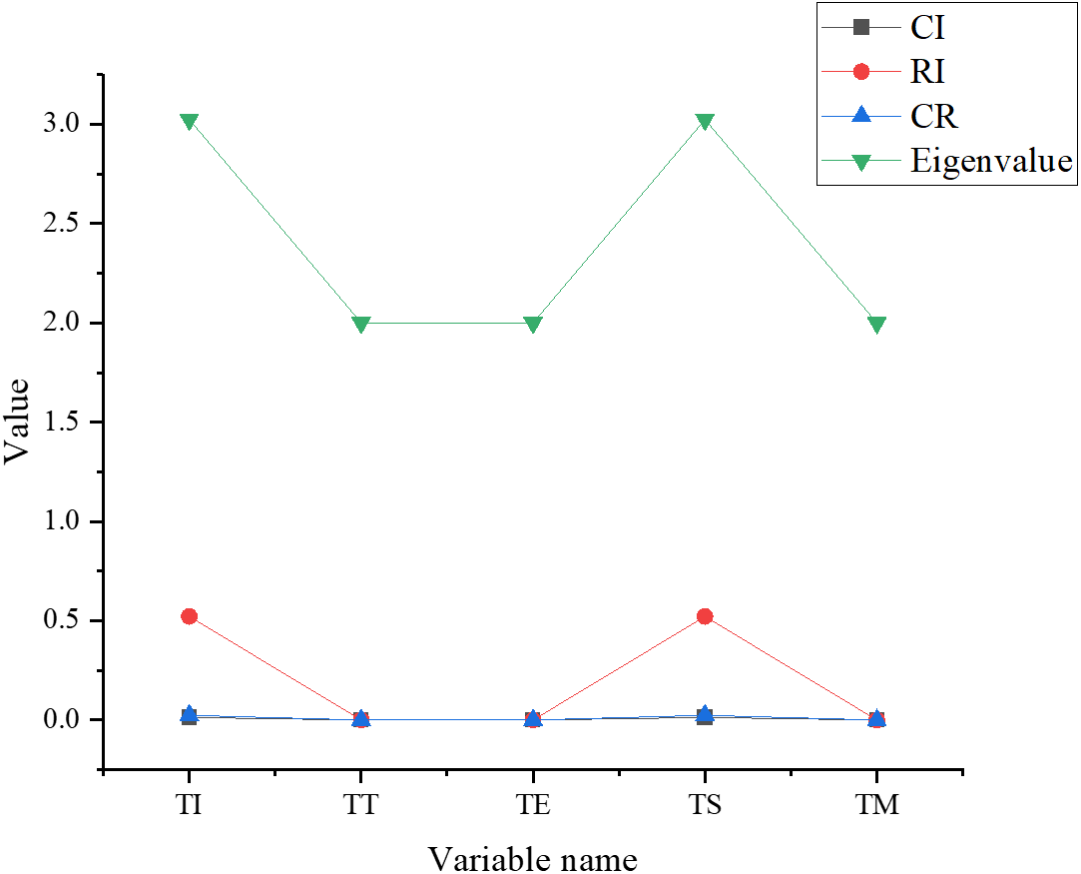
The consistency test results of rural revitalization indexes.

The consistency test results of rural revitalization indexes are indicated in Fig 7.

Fig 7 shows that the CI, CR, and eigenvalue of tourism information (TI) and tourism safety (TS) services are 0.012, 0.023, and 3.024, and consistency test results are less than 0.1. The eigenvalue, CI, and CR of tourism transportation (TT), tourism environment (TE), and tourism management (TM) services are consistent, where CI and CR are about equal to 0, the eigenvalue is 2, and the consistency test result is smaller than 0.1. Overall, the five indexes of rural revitalization align with the consistency test, the data is reliable, and the subsequent evaluation results can be expressed according to the ranking weight vector.

**Fig 8:**
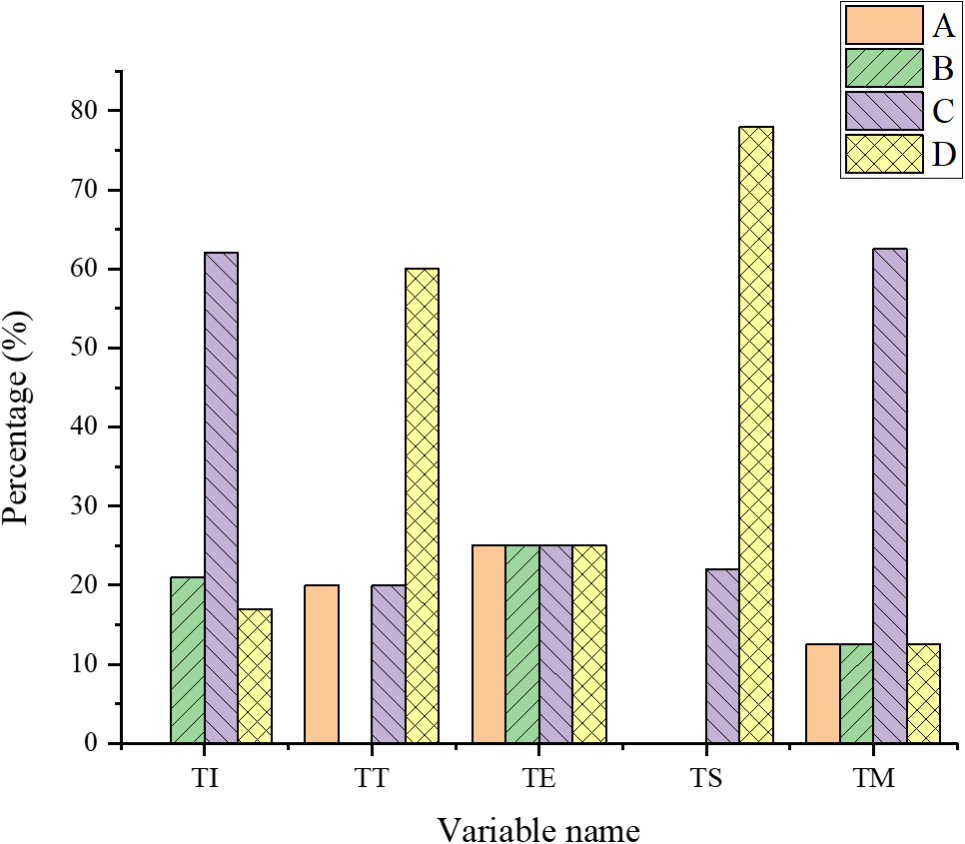
The evaluation results of rural revitalization tourism services.

The evaluation results of rural revitalization tourism services from the angle of a smart city are portrayed in Fig 8.

Fig 8 signifies that TI service has the lowest proportion of Grade A evaluations, and the largest proportion of Grade C, with 62%. Moreover, the proportion of its Grade B evaluations is slightly higher than that of Grade D. In TT services, the Grade B evaluations have the lowest proportion, and Grade D has the largest, which is 60%, and the proportion of Grade A is consistent with that of Grade C. The proportion of evaluations at Grade A, B, C, and D of TE services is consistent. TS service has the largest proportion of Grade A evaluations, 78%, Grade C evaluations account for 22%, and the proportion of Grade A and B evaluations is consistent. TM service has the largest proportion of Grade C evaluations, at 62.5%, and the proportion of A, B and D evaluations is the same. Overall, TI and TM services are majorly evaluated at Grade C. TT services and TS services are principally evaluated at Grade D. TE service does not have outstanding evaluation grades, and the situations at each grade are consistent.

**Fig 9:**
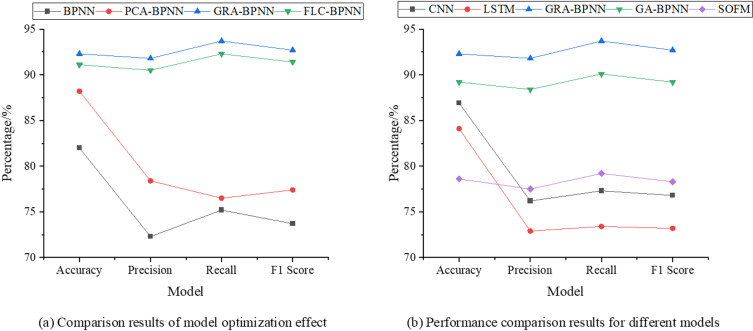
Comparative results of different NNs in rural revitalization evaluation.

Fig 9 presents the comparative results of GRA-BPNN with CNN [45], LSTM Network [46], GAN [47], GA-BPNN [48], and SOFM [49] in rural revitalization evaluation.

In Fig 9(a), the GRA-BPNN model outperforms all other models across all performance metrics. Specifically, GRA-BPNN achieves an accuracy of 92.3%, precision of 91.8%, recall of 93.7%, and an F1 score of 92.7%, significantly surpassing the other models in terms of accuracy, precision, recall, and F1 score. In comparison, the Fuzzy Logic Controller - BPNN (FLC-BPNN) model, while slightly inferior to GRA-BPNN, still outperforms both BPNN and PCA-BPNN. FLC-BPNN achieves an accuracy of 91.1%, precision of 90.5%, recall of 92.3%, and an F1 score of 91.4%, demonstrating strong overall performance, especially in balancing precision and recall. Although PCA-BPNN reduces data dimensionality through PCA, its performance is still below that of GRA-BPNN and FLC-BPNN, with an accuracy of 88.2%, precision of 78.4%, recall of 76.5%, and F1 score of 77.4%. The BPNN model performs the worst, with an accuracy of just 82%, precision of 72.3%, recall of 75.2%, and F1 score of 73.7%, indicating its relatively poor performance in handling complex problems. Overall, the GRA-BPNN model has a clear advantage in processing complex data, providing the best prediction performance. The FLC-BPNN, by incorporating fuzzy logic control, enhances the model’s adaptability and robustness, offering more stable performance in certain applications, particularly in achieving a good balance between precision and recall. PCA-BPNN and BPNN, on the other hand, demonstrate moderate and weak performance, respectively, making them suitable for relatively simple data processing tasks.

In Fig 9(b), the GRA-BPNN model outperforms CNN, LSTM, GA-BPNN, and SOFM models across all performance metrics. In terms of accuracy, GRA-BPNN reaches 92.3%, significantly higher than CNN’s 86.9%, LSTM’s 84.1%, GA-BPNN’s 89.2%, and SOFM’s 78.6%. GRA-BPNN also shows a clear advantage in precision, achieving 91.8%, compared to CNN’s 76.2%, LSTM’s 72.9%, GA-BPNN’s 88.4%, and SOFM’s 77.5%. In terms of recall, GRA-BPNN reaches 93.7%, also higher than CNN’s 77.3%, LSTM’s 73.4%, GA-BPNN’s 90.1%, and SOFM’s 79.2%. Considering both accuracy and recall in the F1 score, GRA-BPNN remains the leader with an F1 score of 92.7%, while CNN scores 76.8%, LSTM 73.2%, GA-BPNN 89.2%, and SOFM 78.3%. In summary, the optimized model in this study demonstrates significant improvements in performance, outperforming other algorithms.

**Fig 10.**
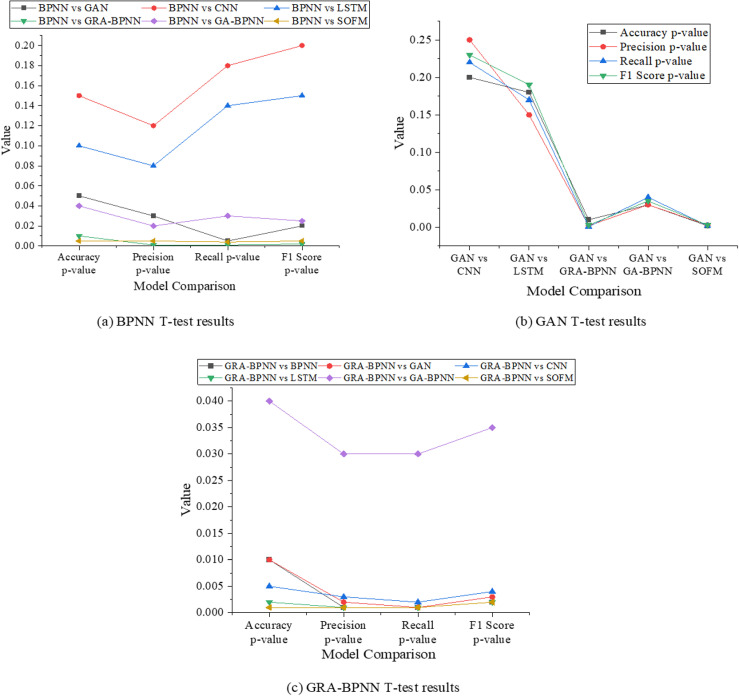
T-test results.

This study uses a t-test to evaluate whether the performance differences between GRA-BPNN and the other models are statistically significant. The specific results are shown in Fig 10.

In Fig 10, the GRA-BPNN model demonstrates significant improvements in performance metrics such as accuracy, precision, recall, and F1 score compared to other baseline models (e.g., BPNN, GAN, CNN, LSTM). GRA-BPNN significantly outperforms BPNN (p = 0.01), GAN (p = 0.01), CNN (p = 0.005), and LSTM (p = 0.002) in terms of accuracy, indicating better prediction accuracy. In terms of precision, GRA-BPNN shows a marked advantage over the other models, particularly compared to BPNN (p = 0.001), GAN (p = 0.002), and CNN (p = 0.003). This suggests that the GRA algorithm improves model precision by effectively reducing false positive predictions. For recall, GRA-BPNN similarly excels, with significant improvements over BPNN (p = 0.001), GAN (p = 0.001), CNN (p = 0.002), and LSTM (p = 0.001), indicating that it is more sensitive in identifying all positive samples. Finally, in terms of the F1 score, GRA-BPNN shows its strong overall performance, with statistically significant differences compared to BPNN (p = 0.002), GAN (p = 0.003), CNN (p = 0.004), and LSTM (p = 0.002). This suggests that GRA-BPNN not only improves accuracy and precision but also maintains a high recall rate, giving it a clear advantage in overall performance. Compared to GA-BPNN (p = 0.035) and SOFM (p = 0.002), the differences are smaller. However, they are still statistically significant, further proving that the improvements made by GRA-BPNN are meaningful and not due to randomness. Therefore, GRA-BPNN exhibits significant advantages across multiple performance metrics, demonstrating the stability and reliability of its optimization effects.

**Fig 11:**
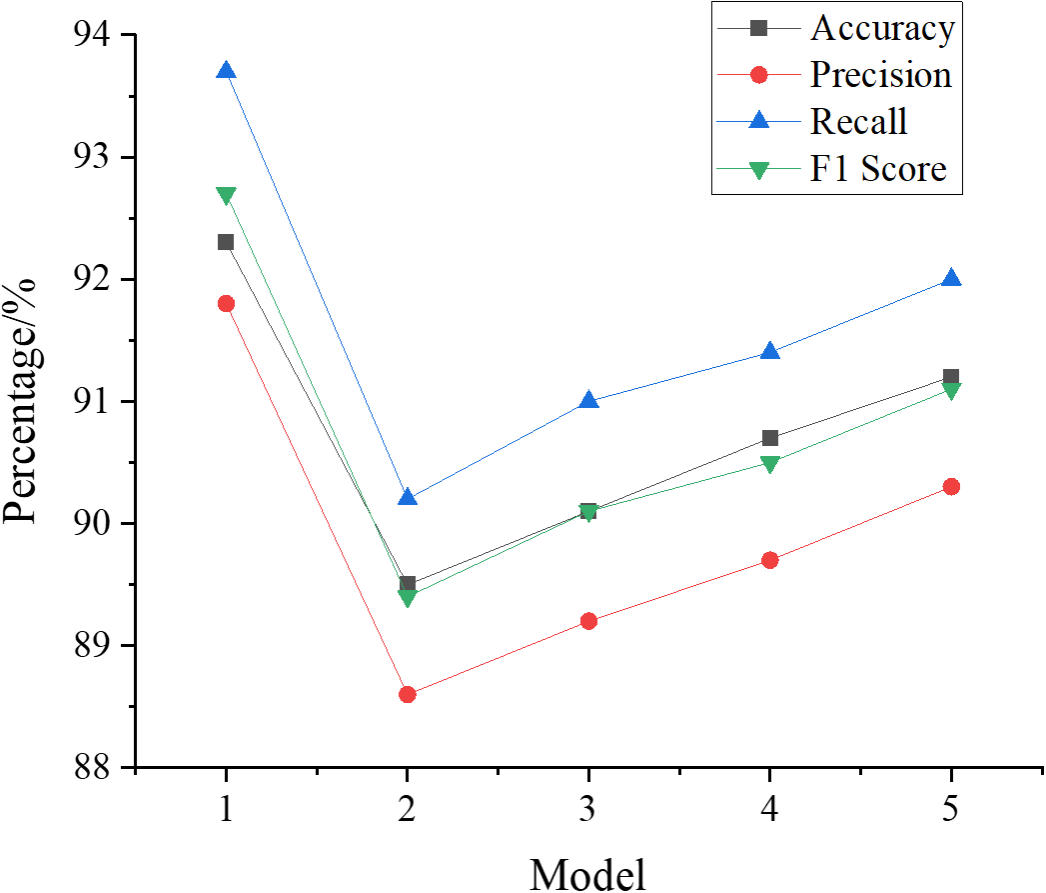
Performance comparison results of the ablation model with the full model.

This study employs a stepwise removal method for different key metrics to assess the performance of the model after removing each individual metric. In the experiment, a GRA-BPNN model containing all metrics is first trained, referred to as Model 1. Then, each metric is removed one by one, resulting in ablation models 2 to 5 (after removing tourism safety, tourism information, tourism management, and tourism transportation, respectively). These models are trained and evaluated on the same dataset. The comparison of the results of the models after removing a specific metric with the complete model in terms of accuracy, precision, recall, and F1 score helps verify the effectiveness of the GRA algorithm in simplifying redundant metrics. The specific comparison results are shown in Fig 11.

In Fig 11, the complete GRA-BPNN model achieves an accuracy of 92.3%, precision of 91.8%, recall of 93.7%, and an F1 score of 92.7%, performing best across all four metrics. In contrast, the model performance decreases when individual metrics are removed. In Model 2 (after removing the tourism safety metric), the accuracy drops to 87.6%, precision to 85.4%, recall to 88.1%, and F1 score to 86.7%, indicating that tourism safety has a significant impact on model performance. In Model 3 (after removing the tourism information metric), the accuracy is 89.2%, precision 87.5%, recall 89.4%, and F1 score 88.4%, showing a slight decrease compared to the complete model. In Model 4 (after removing the tourism management metric), the accuracy is 90.1%, precision 89.0%, recall 90.3%, and F1 score 89.6%, showing a performance decline, but not significant. In Model 5 (after removing the tourism transportation metric), the accuracy is 91.0%, precision 90.2%, recall 91.5%, and F1 score 90.8%, indicating relatively good performance, but still lower than the complete model. These results demonstrate that the GRA algorithm plays a crucial role in retaining key metrics and enhancing model performance, further emphasizing the necessity and importance of multidimensional indicators in tourism evaluation. Through the ablation study, the varying contributions of each metric to the model can be observed, thereby facilitating a better understanding and optimization of the model.

## 5. Discussion

This study conducts an in-depth analysis of rural revitalization issues using the GRA-BPNN model and compares it with other traditional models. The results indicate that GRA-BPNN has significant advantages in rural revitalization assessment, particularly in terms of accuracy, precision, recall, and F1 score. The GRA algorithm plays an important role in this study by effectively simplifying the original complex indicator system through dimensionality reduction techniques. By performing GRA on multiple key indicators, the GRA algorithm extracts the most important factors influencing rural revitalization, reduces the interference of redundant information, and decreases the risk of model overfitting. This advantage allows the GRA-BPNN model to maintain high accuracy while improving computational efficiency and enhancing model stability. Furthermore, the GRA algorithm further optimizes the allocation of indicator weights, ensuring that services from different domains are reasonably reflected in the model, thus providing a more comprehensive evaluation and decision-making support for rural revitalization. Compared to other traditional models, the performance of the model in this study improves significantly. GRA-BPNN outperforms other models in key metrics such as accuracy, precision, recall, and F1 score. Specifically, compared to the PCA-BPNN model, GRA-BPNN demonstrates better feature extraction ability during the data dimensionality reduction process, enabling it to capture the relationships between indicators in rural revitalization more effectively, thus improving performance while reducing model complexity. Compared to the FLC-BPNN model, GRA-BPNN handles nonlinear relationships more accurately and allocates weights to different indicators more reasonably, which contributes to superior performance across all metrics. However, despite the outstanding performance of GRA-BPNN in this study, the superiority of the model is also limited by the size and quality of the dataset. BPNN itself is highly efficient on small datasets, but as the data volume increases, the performance of BPNN may be challenged, especially when dealing with more complex nonlinear relationships. Therefore, although the dataset in this study is moderate in size, in larger datasets, the BPNN model may face the risk of overfitting, leading to increased computation time and performance degradation. In such cases, considering the use of DL models or ensemble learning methods would better address the challenges posed by big data and further improve the model’s generalization ability. In addition, the rural revitalization dataset used in this study is of moderate size, and the BPNN model trains efficiently and achieves good predictive results within this scale. However, as the dataset size expands, the training time and computational resource requirements for the BPNN model will increase exponentially. This is due to the fact that BPNN’s training process relies on numerous weight updates and error feedbacks, and on large datasets, the model may struggle to maintain high predictive accuracy. To improve performance in the context of big data, other algorithms such as deep neural networks, CNN, or ensemble learning-based models can be considered. These methods are generally better at handling complex nonlinear relationships and enhancing the processing capabilities for large-scale datasets. In this study, certain services, such as tourism transportation and safety, receive relatively low scores, which may be attributed to the specificity and complexity of these indicators. The evaluation of tourism transportation typically depends on multidimensional indicators such as regional traffic network density, transportation infrastructure development, and the frequency of traffic accidents. A single indicator often fails to fully reflect the quality of transportation services. Factors like traffic smoothness, the effectiveness of traffic management, and traffic safety significantly impact transportation assessments in rural revitalization. However, these factors are not adequately covered in the existing model, leading to relatively low scores for this indicator. Safety assessments face similar challenges. In rural revitalization, safety not only involves public security but also factors like natural disasters and social safety indices. The variations in these factors are often closely related to socioeconomic conditions, policy environments, and external factors. Therefore, a more detailed design of indicators and richer data support are required in the modeling process. To improve the evaluation accuracy of services such as tourism transportation and safety, future research could incorporate additional indicators to comprehensively reflect the safety situation in rural revitalization. Overall, this study provides a powerful tool for the precise assessment and decision support of rural revitalization, while also pointing out future directions for research development.

## 6. Conclusions

### 6.1 Research contribution

To improve the development level of rural tourism and promote rural revitalization, this study employs BPNN to construct a training model and integrates the GRA algorithm to classify the rural revitalization development of 10 different cities. The rural revitalization indicators are analyzed through the consistency test, and the order of the judgment matrix and the consistency test results of each index are calculated. Ultimately, BPNN is used to construct a tourism service evaluation model, and the evaluation and analysis of rural revitalization tourism from the perspective of a smart city are carried out. In addition, the accuracy of GRA-BPNN evaluation has been confirmed through comparison with algorithms such as LSTM, GAN, and CNN in rural revitalization assessment. The research results demonstrate that: (1) the ten cities’ training results and expected values are relatively consistent, with a larger proportion of excellent and good cities, and the overall development of rural revitalization is good; (2) The five major indicators’ consistency test results of tourism information, security, transportation, environment, and management services are all less than 0.1, which meets the consistency test and confirms the reliability of the research data, laying a foundation for subsequent evaluation of tourism services; (3) Tourism information and management services are mainly C-level evaluations, with 62% and 62.5% respectively. Tourism transportation and safety services are mainly D-level evaluations, with 60% and 78%. The model can represent the level of rural revitalization tourism services based on the perspective of smart cities, providing a basis and direction for the future development of rural tourism; (4) By comparing the GRA-BPNN model with other common NN models, it can be seen that when dealing with small-scale and medium-sized rural revitalization data, the parameters of GRA-BPNN perform well and the evaluation effect is the best.

### 6.2 Future works and research limitations

The shortcomings of this study are as follows. Firstly, in terms of selecting indicators for rural revitalization, combining the situation of rural practical tourism and expert questionnaire surveys can better reflect actual needs. By combining with the actual situation, the elements of rural revitalization can be more comprehensively understood, to optimize evaluation indicators, make them more in line with the actual situation, and improve the practicality and accuracy of the evaluation. Secondly, in NN models, increasing the number of training iterations and reducing training error values are very effective ways to improve. Increasing the number of training sessions can make the model better adapt to data, improve its generalization ability, and thus enhance the accuracy of classification and evaluation. Further reducing the training error value can help improve the model’s stability and reliability. These improvements will help to more accurately evaluate the level of tourism services for rural revitalization, and provide more targeted suggestions and decision-making support for rural revitalization. Finally, this study has not yet applied the model to specific software. In the future, it is possible to further explore rural practice data, strengthen professional investigations related to practical problems, continuously improve models and indicator systems, and enhance the scientific and practical nature of research. Based on the needs of actual software development, the BPNN model is integrated into the software of the rural revitalization service platform to achieve more flexible and efficient decision support tools, thus better serving the practical needs of rural revitalization. Moreover, the successful application of this model in the context of rural revitalization in China demonstrates its effectiveness under specific socioeconomic conditions. However, the generalizability of the model to countries with significantly different socioeconomic conditions from China needs to be carefully evaluated. Different countries exhibit notable differences in resource allocation, infrastructure, policy environments, and cultural backgrounds, which may affect the model’s effectiveness and applicability. Therefore, prior to its application in other countries, comprehensive localization adjustments and validation are necessary to ensure the model can adapt to and effectively address the rural revitalization challenges in those countries. Additionally, cross-national comparative studies and collaborations can provide more experience and data support, further enhancing the model’s generalizability and reliability.

## References

[1] Lai CS, Jia Y, Dong Z, Wang D, Tao Y, Lai QH, et al. A review of technical standards for smart cities. Clean Technol. 2020;2(3):290–310. doi: 10.3390/cleantechnol2030019

[2] Yigitcanlar T, Kankanamge N, Vella K. How are smart city concepts and technologies perceived and utilized? a systematic geo-twitter analysis of smart cities in Australia. J Urban Tech. 2020;28(1–2):135–54. doi: 10.1080/10630732.2020.1753483

[3] Zhao T, Feng Y, Yan M, Liu H, Basodi S. A survey on algorithms for intelligent computing and smart city applications. Big Data Mining and Analytics. 2021;4(3):155–72.

[4] Chen M, Zhou Y, Huang X, Ye C. The integration of new-type urbanization and rural revitalization strategies in China: origin, reality and future trends. Land. 2021;10(2):207.

[5] Yan H, Bun K, Xu S. Rural revitalization, scholars, and the dynamics of the collective future in China. J Peasant Studies. 2021;48(4):853–74.

[6] Han B. Coordinated development in rural revitalization and new urbanization. China Economic Transition= Dangdai Zhongguo Jingji Zhuanxing Yanjiu. 2022;5(1):122–130.

[7] Zhang X, Zhang Z. How do smart villages become a way to achieve sustainable development in rural areas? Smart village planning and practices in China. Sustainability. 2020;12(24):10510. doi: 10.3390/su122410510

[8] Liu J, Zhang Q, Zeng X. Research on the development of city financial systems under the rural revitalization strategy. J Contemp Educ Res. 2021;5(5):14–9.

[9] Zhang R, He Y, Cui W, Yang Z, Ma J, Xu H, et al. Poverty-returning risk monitoring and analysis of the registered poor households based on bp neural network and natural breaks: a case study of Yunyang District, Hubei Province. Sustainability. 2022;14(9):5228.

[10] Guo C, Zhang Y, Liu Z, Li N. A coupling mechanism and the measurement of science and technology innovation and rural revitalization systems. Sustainability. 2022;14(16):10343.

[11] Zhao L, Ran J, Yu G. Research on the implementation path of rural revitalization strategy based on computer big data and industrial revitalization. J Phy: Conf Ser. 2020;1648(2):022165.

[12] Jun H. How to promote rural revitalization via introducing skilled labor, deepening land reform and facilitating investment? China Agric Econ Rev. 2020;12(4):577–82.

[13] Chen L. Driving factors, effect analysis and countermeasures of the development of china’s live broadcast platform. China Finan Econ Rev. 2021;10(1):102–16.

[14] Cen T, Lin S, Wu Q. How does digital economy affect rural revitalization? the mediating effect of industrial upgrading. Sustainability. 2022;14(24):16987. doi: 10.3390/su142416987

[15] García Fernández C, Peek D. Connecting the smart village: a switch towards smart and sustainable rural-urban linkages in Spain. Land. 2023;12(4):822. doi: 10.3390/land12040822

[16] Zhao Y, Li R. Coupling and coordination analysis of digital rural construction from the perspective of rural revitalization: a case study from Zhejiang Province of China. Sustainability. 2022;14(6):3638. doi: 10.3390/su14063638

[17] Zhu M, Jang WS, Pan YH. Research on intelligent space design of smart rural-focus on Xikou village, Zhejiang Province, China. J Korea Convergence Soc. 2022;13(4):245–59.

[18] Yu S, Yang L, Yao D. Research on the development path of smart tourism in Zhangye two-aerospace characteristic Town under the background of rural revitalization. IOP Conf Ser: Earth Environ Sci. 2020;580(1):012064.

[19] Gao Y, Meng Y. Study on construction and evaluation method of eco-environment index system for regional economic development. Fresenius Environmental Bulletin. 2021;30(4):3394–401.

[20] Du Y, Zhao R. Early warning of poverty returning against the background of rural revitalization: a case study of two counties in Guangxi Province, China. Agriculture. 2023;13(5):1087.

[21] Wu L, Zhou J, Li Z. Applying of GA-BP neural network in the land ecological security evaluation. IAENG Int J Computer Sci. 2020;47(1):11–8.

[22] Li S, Fan Z. Evaluation of urban green space landscape planning scheme based on PSO-BP neural network model. Alexandria Engin J. 2022;61(9):7141–53.

[23] Juan YK, Hsu YC. Application of association rules and an artificial neural network to predict the urban development of regional revitalization. J Urban Plann Dev. 2022;148(4). doi: 10.1061/(asce)up.1943-5444.0000876

[24] Muhammad H, Lee H. Prediction of land use and land cover changes for north Sumatra, Indonesia, using an artificial-neural-network-based cellular automaton. Sustainability. 2019;11(11):3024. doi: 10.3390/su11113024

[25] Zhou Y, Shen Y, Yang X, Wang Z, Xu L. Where to revitalize, and how? A rural typology zoning for China. Land. 2021;10(12):1336.

[26] Lu H, Bao J. Spatial differentiation effect of rural logistics in urban agglomerations in china based on the fuzzy neural network. Sustainability. 2022;14(15):9268. doi: 10.3390/su14159268

[27] Sun B, Pan H, Shao S. Countermeasures for improving rural living environments under the background of a rural revitalization strategy based on computer virtualization technology. Sustainability. 2023;15(8):6699. doi: 10.3390/su15086699

[28] Li Y, Xu W, Chen H, Jiang J, Li X. A novel framework based on mask R-CNN and histogram thresholding for scalable segmentation of new and old rural buildings. Remote Sensing. 2021;13(6):1070.

[29] Zhang W, Zhang L. On rural typologies with neural network method: Case study on Xining region. J Regional City Plan. 2020;31(1):12–24.

[30] Hou H, Tang K, Liu X, Zhou Y. Application of artificial intelligence technology optimized by deep learning to rural financial development and rural governance. J Glob Inf Manag. 2021;30(7):1–23.

[31] Xian W, Shang G, Liu Q, Liu Y. Evaluation of rural tourism land competitiveness based on neural-network method and weighted model: a case study of Miyun District in Beijing, China. Acta Agriculturae Zhejiangensis. 2021;33(8):1519.

[32] Gao S, Yang X, Long H, Zhang F, Qin X. The sustainable rural industrial development under entrepreneurship and deep learning from digital empowerment. Sustainability. 2023;15(9):7062.

[33] Haque AB, Bahalul B, Dhiman G. Conceptualizing smart city applications: requirements, architecture, security issues, and emerging trends. Expert Systems 2022;39(5):e12753.

[34] Hartawan MS, Putra AS, Muktiono A. Smart city concept for integrated citizen information smart card or ICISC in DKI Jakarta. Int J Sci Technol Manag. 2020;1(4):364–70.

[35] Liu L, Cao C, Song W. Bibliometric analysis in the field of rural revitalization: current status, progress, and prospects. Int J Environ Res Public Health. 2023;20(1):823. doi: 10.3390/ijerph20010823 36613143 PMC9819429

[36] Jun H. Prioritizing agricultural, rural development and implementing the rural revitalization strategy. China Agr Econ Rev. 2020;12(1):14–9.

[37] Shi J, Yang X. Sustainable development levels and influence factors in rural China based on rural revitalization strategy. Sustainability. 2022;14(14):8908.

[38] Löfving L, Kamuf V, Heleniak T, Weck S, Norlén G. Can digitalization be a tool to overcome spatial injustice in sparsely populated regions? The cases of digital Västerbotten (Sweden) and smart country side (Germany). European Planning Studies. 2022;30(5):917–34.

[39] Khan F, Ali Y. A facilitating framework for a developing country to adopt smart waste management in the context of circular economy. Environ Sci Pollut Res Int. 2022;29(18):26336–51. doi: 10.1007/s11356-021-17573-5 34850345 PMC8632210

[40] Meyer JG. Deep learning neural network tools for proteomics. Cell Rep Methods. 2021;1(2):100003. doi: 10.1016/j.crmeth.2021.100003 35475237 PMC9017218

[41] Mittal S. A survey of FPGA-based accelerators for convolutional neural networks. Neural Comput Appl. 2020;32(4):1109–39.

[42] Zhang Y, Tang J, Liao R, Zhang M, Zhang Y, Wang X, et al. Application of an enhanced BP neural network model with water cycle algorithm on landslide prediction. Stoch Environ Res Risk Assess. 2021;35(6):1273–91.

[43] Chen G. Security precautionary technology for enterprise information resource database based on genetic algorithm in age of big data. J ComputMethods Sci Engin. 2020;20(2):427–34.

[44] Yuan M, Li C. Research on global higher education quality based on BP neural network and analytic hierarchy process. JCC. 2021;09(06):158–73. doi: 10.4236/jcc.2021.96009

[45] Liu Y, Shu B, Chen Y, Zhang H. Spatial vulnerability assessment of rural settlements in hilly areas using BP neural network algorithm. Ecological Indicators. 2023;157:111278. doi: 10.1016/j.ecolind.2023.111278

[46] Lee C, Lee J, Park S. Forecasting the urbanization dynamics in the Seoul metropolitan area using a long short-term memory–based model. Environ Plan B: Urban Analy City Sci. 2023;50(2):453–68.

[47] Adamiak M, Będkowski K, Bielecki A. Generative adversarial approach to urban areas’ NDVI estimation: a case study of Łódź, Poland. Quaestiones Geographicae. 2023;42(1):87–106.

[48] Wang S, Han M. Rural revitalization evaluation using a hybrid method of BP neural network and genetic algorithm based on deep learning model. IJACSA. 2024;15(2). doi: 10.14569/ijacsa.2024.0150221

[49] Ji J, Huang A, Liu C, Dai L. The application of the SOFM neural network and Internet of Things in rural revitalization. IEEE Access. 2023;11(1):119427–35.

